# Increasing incidence of adenocarcinoma of the gastric cardia and adjacent sites.

**DOI:** 10.1038/bjc.1990.314

**Published:** 1990-09

**Authors:** J. Powell, C. C. McConkey

**Affiliations:** Regional Cancer Registry, Queen Elizabeth Medical Centre, Birmingham, UK.

## Abstract

Data in a regional cancer registry covering a population of 5 million and with an efficiency of registration of over 95% have been used to examine incidence trends in oesophageal and gastric carcinoma. In the West Midlands Region of the UK, during the period 1962 to 1981 the age standardised incidence of gastric carcinoma decreased by 20%. However, an analysis by both histological type and detailed site reveals that while the incidence of distal lesions is diminishing, the incidence of adenocarcinoma of the oesophagus and cardia is increasing. The proximal and distal lesions also exhibit marked differences in social class distribution and sex ratio. The results strongly suggest that the aetiological factors involved for cardia and adjoining sites are different from those for pyloric antrum.


Br. J. Cancer (1990), 62, 440-443 © Macmillan Press Ltd., 1990

### Increasing incidence of adenocarcinoma of the gastric cardia and adjacent sites

J. Powell & C.C. McConkey

*Regional Cancer Registry, Queen Elizabeth Medical Centre, Birmingham B15 2TH, UK.*

**Summary** Data in a regional cancer registry covering a population of 5 million and with an efficiency of registration of over 95% have been used to examine incidence trends in oesophageal and gastric carcinoma. In the West Midlands Region of the UK, during the period 1962 to 1981 the age standardised incidence of gastric carcinoma decreased by 20%. However, an analysis by both histological type and detailed site reveals that while the incidence of distal lesions is diminishing, the incidence of adenocarcinoma of the oesophagus and cardia is increasing. The proximal and distal lesions also exhibit marked differences in social class distribution and sex ratio. The results strongly suggest that the aetiological factors involved for cardia and adjoining sites are different from those for pyloric antrum.

The morbidity and mortality rates of gastric carcinoma have been decreasing in the West Midlands Region of England as they have generally throughout the world. However, mortality rates usually refer to all histological types and all sites combined, and there have been a number of reports (Yang & Davis, 1988; Moller & Moller Jensen, 1988; Antonioli & Cady, 1984; Storm, 1983) suggesting that adenocarcinoma of the cardia may be increasing.

The data available in the West Midlands Regional Cancer Registry enable incidence rates to be calculated for both histological and anatomical subdivisions within organs. The results which are presented here indicate that not all sites are behaving in the same way and, even within sub-sites, there is considerable variation in the trends observed for different histological types. In this paper data have been examined for both oesophagus and stomach.

### Methods

All the analyses here are based on data in the West Midlands Regional Cancer Registry. The Registry has been population-based since 1957 covering a population of just over 5 million. Efficiency of registration is believed to have been over 95% since the early 1960s (Waterhouse, 1974). As part of a comprehensive epidemiological and clinical study of the data in the registry (Fielding *et al.*, 1989), trends in both histological and site distribution were examined for the period 1957-1981. Registration was known to be incomplete during the earliest years and, for this reason, the results given here are restricted to the period 1962-1981 except, for reasons given later, in the analysis by type of first symptom. As part of the original study the data had already been subjected to intensive validity checks and assessments which are described in the monographs on oesophageal and stomach cancer (Matthews *et al.*, 1987; Fielding *et al.*, 1989). In this study all unusual types of cancer have been excluded. In oesophagus, these were defined as transitional cell and basal cell carcinoma, carcinoid, pseudosarcoma, melanoma and sarcoma, and in stomach as adeno-squamous and squamous cell carcinoma, carcinoid, melanoma and sarcoma. The total numbers so excluded were 23 (0.4%) from oesophagus and 136 (0.5%) from stomach.

To facilitate comparisons, the incidence rates have been age standardised to the world standard population (Muir *et al.*, 1987). Site specification and histological verification are clearly related to the extent of investigative procedures and since 1970 there has been a very marked rise in the number of endoscopies. Endoscopy here includes both rigid oesophagoscopy and the flexible tubes which became prevalent in the early 1970s, since the two types cannot be distinguished in the Registry code used. The effect of the rise in patients undergoing endoscopy was examined within each sub-site, these are likely to be underestimates because not all investigations will have been routinely reported to the Registry. The size of this error is not known but is unlikely to have changed substantially over the study period.

Another factor used to assess the validity of the changes in sub-site incidence was the type of first symptom, although this was only available for the period 1957-76. Cardia patients have a very much higher rate of dysphagia than others and an increase in dysphagia rate would, therefore, imply an increase in lesions involving the cardia. However, since the number of all recorded symptoms had increased, a standardised rate based on the all symptoms rate in 1972-76 was calculated for each 5-year period.

Other factors examined were mean age, sex ratio and social class. The study of socio-economic factors was restricted to the period 1966-80 and was based on the 1970 Classification of Occupations (OPCS, 1970). Social classes 1 and 2 are professional or managerial, 3 includes both clerical and skilled manual, and 4 and 5 are semi-skilled or unskilled manual. For males only, the social class distributions observed in each site were compared with those observed in cancers of all organs registered during the same period.

### Results

The age standardised incidence rates given in Table I indicate a slight increase in oesophageal carcinoma, largely as a result of a sharp increase in adenocarcinoma rates. In stomach, since unusual tumours are excluded, only one histological category is used, that is, all those histologically verified. For the purposes of this study, this is termed *adenocarcinoma* but it does include 2097 cases (20.5%) of anaplastic carcinoma and 461 cases (4.5%) of carcinoma, type not further specified. Adenocarcinoma, as so defined, showed a highly significant increase ($P < 0.01$) but those with no histology declined by more than half over the 20 years ($P < 0.001$).

In Table II, adenocarcinoma rates are further analysed by sub-site. The oesophagus rates show a significant increase in the middle and lower thirds ($P < 0.05$). In stomach, cardia and other single sites show marked increases ($P < 0.01$ and $P < 0.001$ respectively). Other single sites comprise lesions specified as greater or lesser curvatures and anterior or posterior surfaces, the numbers in each being too small to analyse separately. Tumours of the pyloric antrum are remarkably constant, whereas body shows some fluctuation. However, the unspecified group has decreased by over one third and this is highly significant ($P < 0.01$). This group

Correspondence: J. Powell.
Received 20 November 1989; and in revised form 11 April 1990.

**Table I** Annual incidence rates for carcinoma of the oesophagus and stomach by histological type

| | Average annual incidence rates (age standardised) per 100,000 in: | | | | P value for trend |
|---|---|---|---|---|---|
| | 1962–66 | 1967–71 | 1972–76 | 1977–81 | |
| Oesophagus | 3.44 | 3.30 | 3.65 | 4.11 | $P<0.05$ |
| | (1169) | (1209) | (1440) | (1693) | |
| Stomach | 19.22 | 17.70 | 16.53 | 15.30 | $P<0.01$ |
| | (6607) | (6510) | (6588) | (6300) | |
| Oesophagus | | | | | |
| Adenocarcinoma | 0.14 | 0.18 | 0.37 | 0.63 | $P<0.05$ |
| | (48) | (64) | (137) | (235) | |
| Anaplastic carcinoma | 0.14 | 0.19 | 0.26 | 0.26 | |
| | (50) | (69) | (101) | (109) | |
| Squamous carcinoma | 1.90 | 1.85 | 2.05 | 2.11 | |
| | (632) | (670) | (794) | (844) | |
| Malignant type not specified | 0.04 | 0.04 | 0.05 | 0.09 | |
| | (13) | (13) | (24) | (47) | |
| No histology | 1.22 | 1.04 | 0.92 | 1.02 | |
| | (426) | (393) | (384) | (458) | |
| Stomach | | | | | |
| Adenocarcinoma | 9.18 | 9.48 | 10.03 | 10.37 | $P<0.01$ |
| | (3001) | (3333) | (3807) | (4062) | |
| No histology | 10.04 | 8.22 | 6.49 | 4.93 | $P<0.001$ |
| | (3606) | (3177) | (2781) | (2238) | |

The total number of patients in each group are given in parentheses.

**Table II** Annual incidence rates for adenocarcinoma of oesophagus and stomach by sub-site

| | Average annual incidence rates (age standardised) per 100,000 in: | | | | P value for trend |
|---|---|---|---|---|---|
| | 1962–66 | 1967–71 | 1972–76 | 1977–81 | |
| Oesophagus | | | | | |
| Upper third | 0.007 | 0.007 | 0.008 | 0.010 | |
| | (3) | (2) | (3) | (3) | |
| Middle third | 0.031 | 0.043 | 0.114 | 0.198 | $P<0.05$ |
| | (11) | (16) | (44) | (73) | |
| Lower third | 0.101 | 0.129 | 0.227 | 0.390 | $P<0.05$ |
| | (33) | (46) | (83) | (144) | |
| Unspecified | 0.003 | 0.000 | 0.020 | 0.032 | |
| | (1) | (0) | (7) | (15) | |
| Stomach | | | | | |
| Cardia | 0.75 | 1.25 | 1.51 | 2.01 | $P<0.01$ |
| | (243) | (428) | (557) | (739) | |
| Pyloric antrum | 2.63 | 2.51 | 2.59 | 2.50 | |
| | (874) | (902) | (1009) | (1026) | |
| Body | 0.97 | 0.97 | 1.43 | 1.34 | |
| | (314) | (342) | (547) | (532) | |
| Other single sites | 1.40 | 1.71 | 2.07 | 2.41 | $P<0.001$ |
| | (447) | (591) | (771) | (923) | |
| Unspecified or multiple | 3.44 | 3.05 | 2.43 | 2.12 | $P<0.01$ |
| | (1123) | (1070) | (923) | (842) | |

The total number of patients in each group are given in parentheses.

includes tumours involving more than one site, for example, body and pyloric antrum.

To counteract the effect of the increased numbers with sub-site specified, the incidence rates for each sub-site were expressed as a proportion of the rate for all specified sites. The results, for each quinquennium, are illustrated in Figure 1. For completeness, the rates by sub-site for stomach cases with no histological verification were also calculated and are given in Table III. The only statistically significant changes are in pyloric antrum and unspecified sites, both of which show a highly significant decrease ($P<0.01$). Oesophagus sub-sites with no histology have not been analysed because of the known high proportion of squamous cell carcinomas.

The increasing use of endoscopy throughout the study period is described in relation to sub-site in Table IV. The very high proportion (65.9%) of cardia diagnosed by 'endoscopy' in the decade 1962–71 is explained by the fact that rigid oesophagoscopy could be used to diagnose lesions of the cardia but not of other gastric sites. In Table V, the dysphagia rates are compared for each sub-site and for all stomach standardised to allow for the increase in the number of all recorded symptoms. The table shows that dysphagia is increasing in the unspecified group ($P<0.001$) and in the all stomach group ($P<0.05$). The mean ages in cardia showed a

**Figure 1** Adenocarcinoma of stomach: sub-site distribution by quinquennium, based on histologically verified cases with a specified site. Cardia ——, Other single sites ----, Pyloric antrum ············, Body ······.

small overall increase but it was not consistent (64.3, 64.5, 66.6 and 66.4 in each of the quinquennia) whereas pyloric antrum showed a bigger and more consistent increase (65.1, 66.8, 68.4 and 70.0). The sex ratios for adenocarcinoma of the oesophagus increased in the latest quinquennium (1.8M:1F, 1.8:1 2.3:1 and 2.9:1). The overall sex ratios for cardia and pyloric antrum were markedly different being 2.9M:1F for cardia and 1.4M:1F for pyloric antrum but with very little change over time.

For males, the social class distributions observed in each sub-site were compared with those for all male cancers of any organ registered during the same period. The results given in Table VI show that, for instance, in social classes 1 and 2 there were 16% more cardia cases and 27% less pyloric antrum cases recorded than would be expected if the distribution was the same as that for all sites of cancer. In classes 4 and 5 there were 10% less cardia and 8% more pyloric antrum. These differences are highly significant ($P < 0.001$).

**Table III** Annual incidence rates for sub-sites of stomach for cases with no histological verification

| | *Average annual incidence rates (age standardised) per 100,000 in:* 1962–66 | 1967–71 | 1972–76 | 1977–81 | *P value for trend* |
|---|---|---|---|---|---|
| Stomach | | | | | |
| Cardia | 0.25 | 0.38 | 0.41 | 0.43 | |
| | (89) | (135) | (172) | (182) | |
| Pyloric antrum | 1.23 | 1.16 | 1.05 | 0.92 | $P<0.01$ |
| | (429) | (436) | (458) | (410) | |
| Body | 0.67 | 0.67 | 0.87 | 0.69 | |
| | (229) | (253) | (367) | (319) | |
| Other single sites | 0.70 | 0.76 | 0.64 | 0.54 | |
| | (241) | (289) | (274) | (228) | |
| Unspecified or multiple | 7.19 | 5.25 | 3.52 | 2.36 | $P<0.01$ |
| | (2618) | (2064) | (1510) | (1099) | |

The total number of patients in each group are given in parentheses.

**Table IV** Carcinoma of the stomach: proportions (%) by sub-site, decade and use of endoscopy

| | *Endoscopy ± barium meal* 1962–71 | 1972–81 | *No endoscopy* 1962–71 | 1972–81 |
|---|---|---|---|---|
| Cardia | 65.9 | 33.6 | 11.2 | 12.5 |
| Pyloric antrum | 10.1 | 22.0 | 44.1 | 40.0 |
| Body | 13.4 | 17.2 | 18.8 | 22.4 |
| Other single sites | 10.6 | 27.2 | 25.9 | 25.1 |
| Total with specified site | 100.0 (367) | 100.0 (2775) | 100.0 (6474) | 100.0 (5739) |

**Table V** Carcinoma of the stomach: proportion (%) with dysphagia during the period 1957–76

| | *1957–61* | *1962–66* | *1967–71* | *1972–76* | *P value for trend* |
|---|---|---|---|---|---|
| Cardia | 52.2 | 55.6 | 64.7 | 58.4 | |
| | (267) | (314) | (527) | (682) | |
| Pyloric antrum | 2.7 | 1.7 | 4.5 | 3.5 | |
| | (1079) | (1214) | (1250) | (1348) | |
| Body | 9.2 | 12.9 | 16.2 | 14.2 | |
| | (418) | (514) | (561) | (830) | |
| Other single sites | 2.9 | 3.3 | 5.9 | 5.7 | |
| | (579) | (634) | (800) | (941) | |
| Unspecified or multiple | 4.8 | 6.1 | 7.6 | 8.8 | $P<0.001$ |
| | (2511) | (3043) | (2716) | (2104) | |
| All sites | 7.1 | 8.2 | 12.7 | 13.6 | $P<0.05$ |
| | (4854) | (5719) | (5854) | (5905) | |

The total number of patients in each group with symptoms specified, are given in parentheses.

**Table VI** Carcinoma of stomach and adenocarcinoma of oesophagus, males, 1966–80: distribution (%) by social class

| *Social class* | *Oesophagus adenocarcinoma* | *Stomach* Cardia | *Pyloric antrum* | *Body and other sites* | *Unspecified site* | *Cancer of any organ* |
|---|---|---|---|---|---|---|
| I | 4.7 | 2.8 | 1.0 | 1.6 | 1.5 | 2.3 |
| II | 17.0 | 15.7 | 10.4 | 11.3 | 11.6 | 13.7 |
| III | 51.4 | 51.0 | 52.0 | 50.5 | 49.1 | 50.2 |
| IV | 19.8 | 22.3 | 23.2 | 24.5 | 25.7 | 23.7 |
| V | 7.1 | 8.2 | 13.4 | 12.1 | 12.1 | 10.1 |
| Total with known social class | 100.0 (253) | 100.0 (1490) | 100.0 (2129) | 100.0 (3116) | 100.0 (4093) | 100.0 (111449) |
| Social class not known | (21) | (80) | (205) | (289) | (351) | (23002) |

The total number of patients whose social classes were known and unknown are given in parentheses.

**Discussion**

The increase in the incidence of carcinoma of the cardia over the study period is at variance with the widely reported decrease in stomach carcinoma as a whole. Evaluating this increase was complicated by the rapid changes in investigative procedures and the marked effect these had on the number with histological confirmation and specified site. Thus in Table I, although the overall incidence of stomach carcinoma has decreased by 20%, those with no histology have decreased by 51%. The corresponding rise in cases with histological confirmation was only 13%, confirming the overall decline in stomach cancer. However, the increase in cases with histological confirmation was not uniformly distributed throughout the sites, as can be seen in Table II. This Table also shows a further confounding factor resulting from the increased number of investigations, that is, a 38% decrease in the proportion with unspecified site. Could the apparent increase in cardia be simply an artefact of these changes? There are a number of reasons for believing that this is not the explanation:

1 Since the series is from an unselected population with virtually complete registration, the other histologies and sites form a useful control. In oesophagus the incidence of squamous cell carcinoma has remained virtually unchanged, in contrast to adenocarcinoma. In stomach pyloric antrum has either remained constant (histologically verified) or decreased significantly (no histology), in contrast to cardia.
2 The increasing dysphagia rates in the period 1957–76 in the unspecified site group suggest that it contains an increasing proportion of cardia cases.
3 Any site which is decreasing in incidence exhibits an increase in mean age over and above that expected in an ageing population. Cardia shows the least increase of any site.
4 The association of endoscopy usage with cardia tumours may be used to argue that the reported increases in cardia are merely a reflection of increasing endoscopy usage. However, since in the earlier period there were already endoscopic methods in use for the diagnosis of cardia tumours this is unlikely.
5 When the sex ratio and social class distribution are compared, the similarities observed between adenocarcinoma of the oesophagus and gastric cardia are in marked contrast to the differences exhibited between cardia and pyloric antrum.
6 Unpublished data for 1982–85 from the Registry shows that the trends described here are continuing.

Our earlier work (Fielding *et al.*, 1989) had confirmed that cardia has a much poorer prognosis than pyloric antrum, the five year survival of curatively resected cases being 9.7% compared to 23.9%, with a higher operative mortality rate, 26.4% compared to 9.9%. If the changes observed are confirmed and if they apply to other populations, then improvements in survival due to better diagnosis, surgery and anaesthetics will be reduced by the increasing numbers of patients with a poorer prognosis than those in the pyloric antrum.

The results by socio-economic group indicate that the increasing incidence of adenocarcinoma of oesophagus and cardia are unlikely to be uniform throughout the population but are relatively higher in the professional classes (1 and 2). In the United Kingdom, it is these classes who, overall were the first to reduce smoking, which suggests that tobacco is less likely to be a major factor in cancer of the gastric cardia. However, alcohol consumption, particularly of spirits, and dietary habits will obviously vary between socio-economic groups although precise evaluation is notoriously difficult. In summary, we would suggest that any study on the possible relationship between cancer of the stomach and life style should analyse proximal and distal lesions separately to avoid confounding due to different aetiological factors.

This work is based entirely on data from the Birmingham and West Midlands Regional Cancer Registry. The analysis is an extension of our work on Clinical Cancer Monographs which are wholly supported by the Cancer Research Campaign. We are grateful to Mrs Judy Connor and Mrs Margaret Williams for their careful preparation of the text and tables.

**References**

ANTONIOLI, D.A. & CADY, B. (1984). Changing aspects of gastric adenocarcinoma. *N. Engl. J. Med.*, **310**, 1538.

FIELDING, J.W.L., POWELL, J., ALLUM, W.H., WATERHOUSE, J.A.H. & MCCONKEY, C.C. (1989). *Cancer of the Stomach*, (Clinical Cancer Monographs; Vol. 3). The Macmillan Press: London.

MATTHEWS, H.R., WATERHOUSE, J.A.H., POWELL, J., MCCONKEY, C.C. & ROBERTSON, J.E. (1987). *Cancer of the Oesophagus*, (Clinical Cancer Monographs; Vol. 1). The Macmillan Press: London.

MOLLER, H. & MOLLER JENSEN, O. (1988). Trends in incidence of oesophagus, cardia and stomach in Denmark, 1943–1982. *6th Annual ECP Symposium*. Int. Symposium of Gastric Carcinogenesis: London.

MUIR, C., WATERHOUSE, J.A.H., MACK, T., POWELL, J. & WHELAN, S. (eds) (1987). *Cancer Incidence in Five Continents: Vol. 5*. IARC Scientific Publications No. 88: Lyon.

OPCS (1970). *Classification of Occupation*. HMSO: London.

STORM, H.H. (1983). Comparison of the development of cancer of the lungs, larynx, oesophagus and stomach in Denmark during the period 1943–1977. *Ugeskr. Laeger*, **145**, 1178.

WATERHOUSE, J.A.H. (1974). *Cancer Handbook of Epidemiology and Prognosis*. Churchill Livingstone: London.

YANG, P.C. & DAVIS, S. (1988). Epidemiological characteristics of adenocarcinoma of the gastric cardia and distal stomach in the United States, 1973–82. *Int. J. Epidemiology*, **17**, 293.


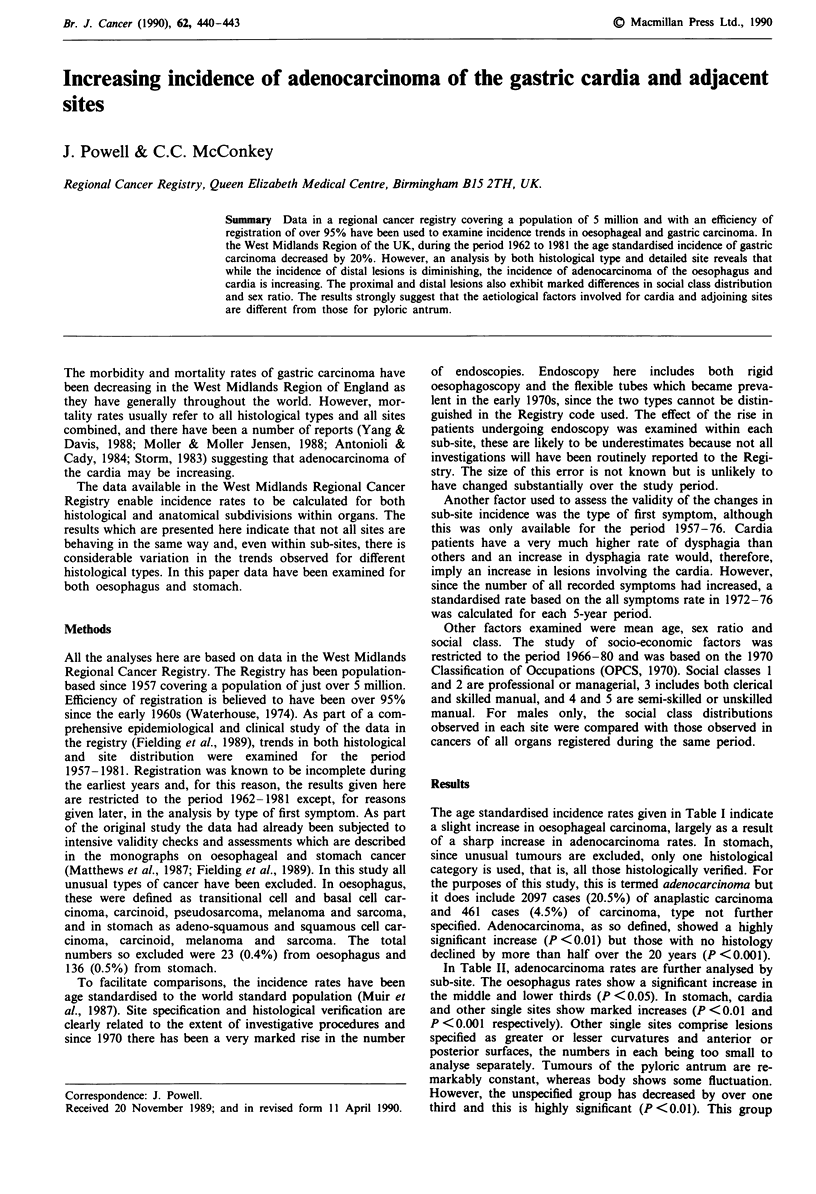

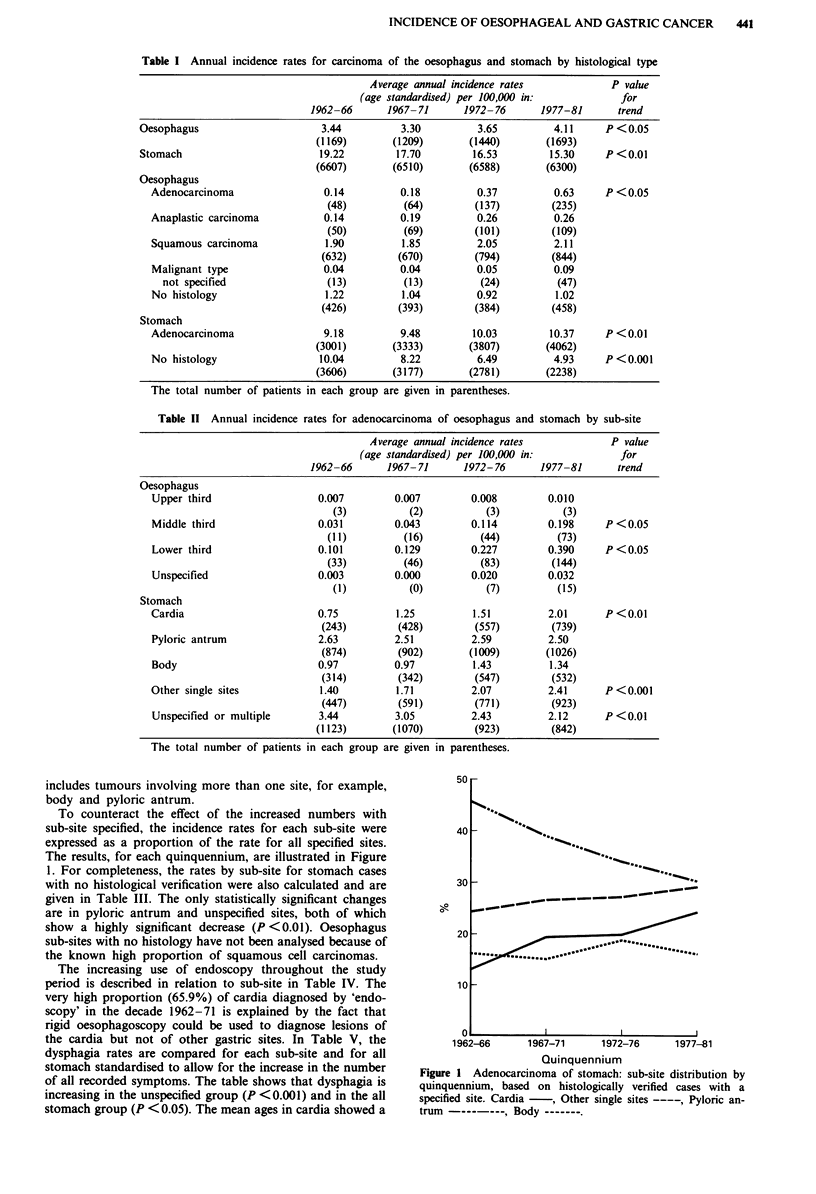

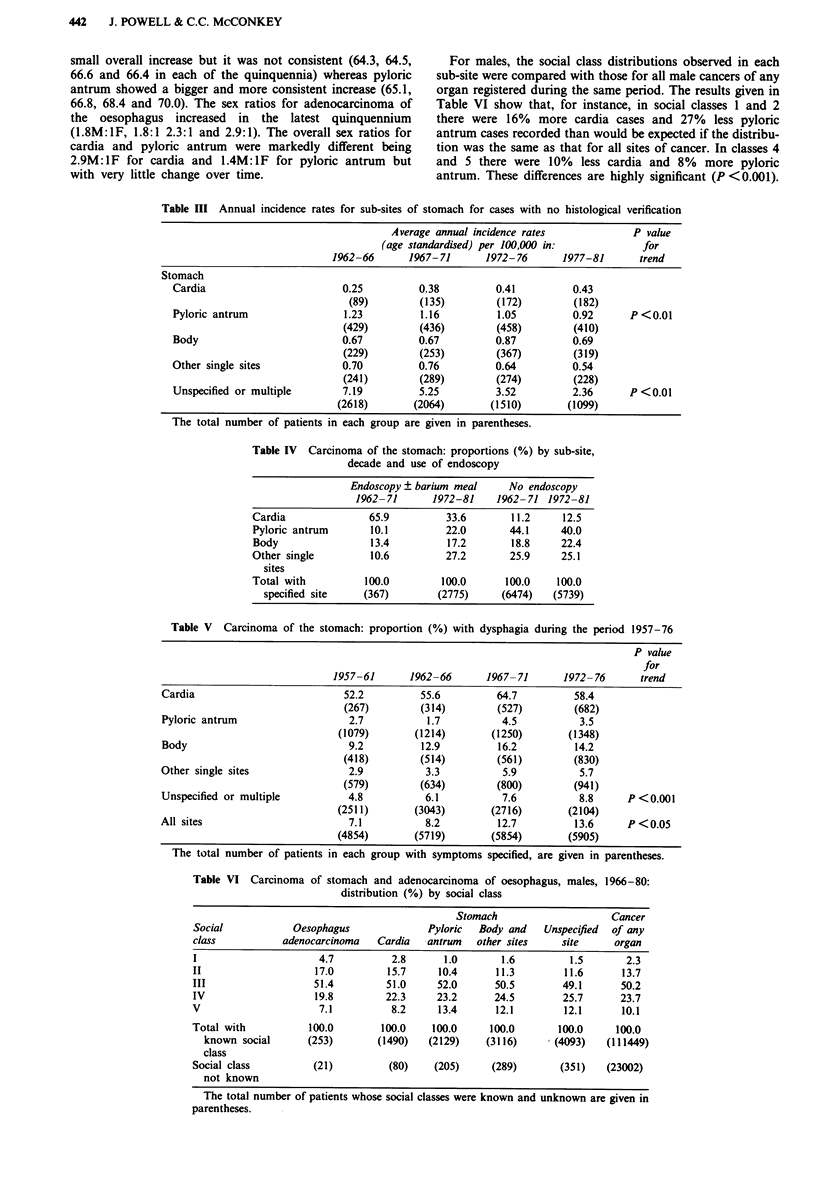

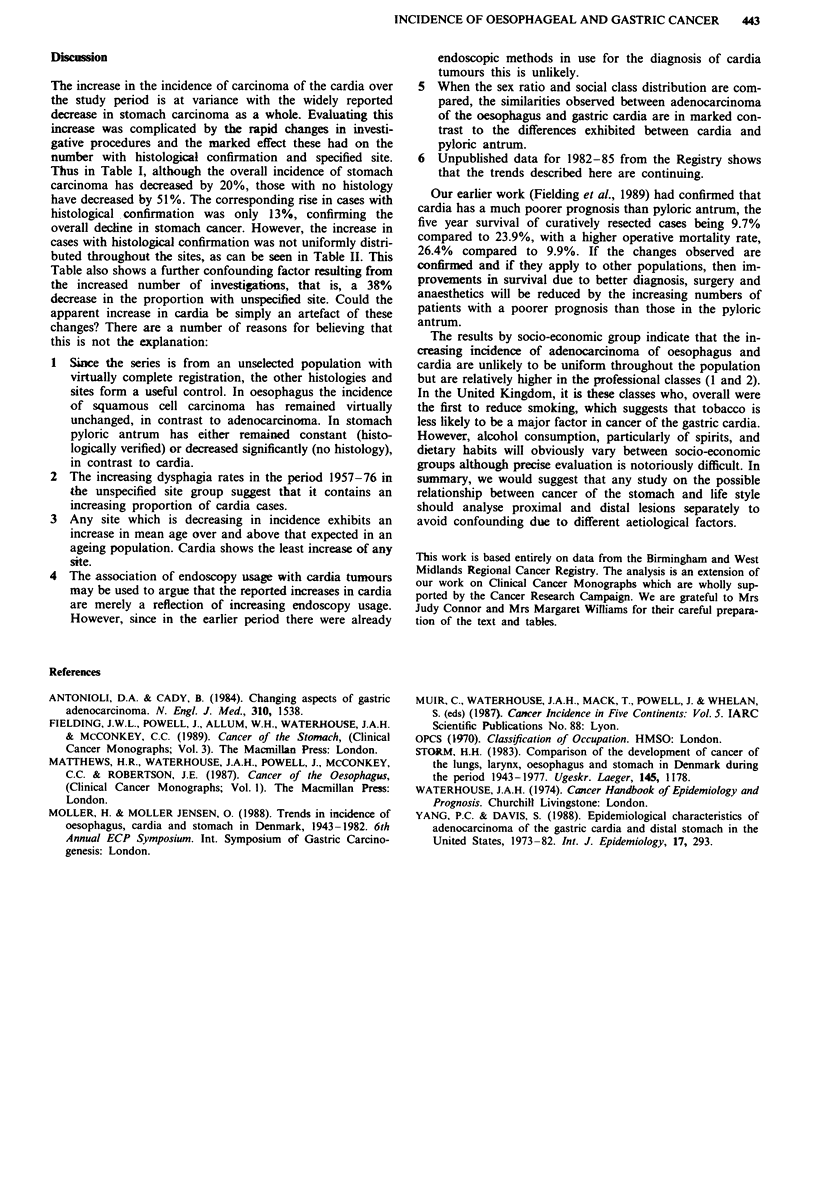


## References

[OCR_00498] Antonioli D. A., Cady B. (1984). Changing aspects of gastric adenocarcinoma.. N Engl J Med.

[OCR_00526] Storm H. H. (1983). Sammenligning of udvikling i lunge-, larynx-, esophagus- og ventrikelcancer 1943-1977 i Danmark.. Ugeskr Laeger.

[OCR_00535] Yang P. C., Davis S. (1988). Epidemiological characteristics of adenocarcinoma of the gastric cardia and distal stomach in the United States, 1973-1982.. Int J Epidemiol.

